# Correlation of index finger length to vertical dimensions of occlusion for edentulous patients and their satisfaction: a randomized controlled trial

**DOI:** 10.1038/s41598-023-33722-x

**Published:** 2023-05-07

**Authors:** Shujah Adil Khan, Syed Murtaza Raza Kazmi, Shahbaz Ahmed, Ume Hani, Ziaullah Choudhry, Adnan Sukkurwala

**Affiliations:** 1 Department of Prosthodontics, Liaquat College of Medicine and Dentistry, Karachi, Pakistan; 2 Section of Dentistry, Department of Surgery, Aga Khan University Hospital, Karachi, Pakistan; 3 Department of Operative Dentistry, Dr. Ishrat-Ul-Ebad Khan Institute of Oral Health Sciences, Dow University of Health Sciences, Karachi, Pakistan; 4 Department of Oral Biology, Dr. Ishrat-Ul-Ebad Khan Institute of Oral Health Sciences, Dow University of Health Sciences, Karachi, Pakistan; 5 Department of Prosthodontics, Dr. Ishrat-Ul-Ebad Khan Institute of Oral Health Sciences, Dow University of Health Sciences, Karachi, Pakistan; 6 Department of Community Dentistry, Dow Dental College, Dow University of Health Sciences, Karachi, Pakistan

**Keywords:** Removable prosthodontics, Occlusion

## Abstract

The prevalence of edentulism is pandemic and people resort to complete dentures for the restoration of missing teeth and esthetics. However, the determination of the correct occlusal vertical dimensions (OVD) constitutes to play an important role in overall patient satisfaction. The objective of this study was to apply anthropometric methods to correlate the length of index finger (2D) to measure the OVD from base of the nose to the base of the chin (Sn–Me) and to assess satisfaction by comparing both the methods. A total of 80 edentulous patients were randomized and controlled for this trial into experimental and control groups. A correlation was found between Sn–Me and finger measurements, dentures’ satisfaction was assessed after a 1-week follow-up and marked according to the Visual Analog Scale. Our findings established that finger measurements are greater among males, and in both genders, positive, and statistically significant correlations exist between the facial and finger length measurements. Moreover, 97.0% patients from experimental group were satisfied with the use of complete dentures through the new anthropometric method. Hence measuring the length of index finger can be an adjunct method for the restoration of OVD and is a relatively time-effective and simple method with a satisfactory follow-up.

*Trial registration*: ID: NCT05153213 (https://clinicaltrials.gov/ct2/show/NCT05153213).

## Introduction

Edentulism by definition is the state of being without teeth^1^, its prevalence varies across the world and in Pakistan, approximately 4.1% of the population above the age of 65 is edentulous with an estimated increase to 9.3% by 2030^2^. Complete dentures will continue to remain the standard treatment option for the edentulous, for replacement of missing teeth and restoration of esthetics impacting their social lives^3^. The provision of complete dentures relies upon the measurement of occlusal vertical dimensions, centric occlusion and arrangement of teeth^4^. Hence, it is imperative to determine the correct occlusal vertical dimension (OVD), and in consonance with ‘The Glossary of Prosthodontic Terms’ is the span amongst two chosen anatomically distinct marks (one on the prominence of the nose and another on the chin) when in inter-cuspal position^1^.

To establish harmony of the lower one-third of the face, the physiologic OVD must be restored and one of the prime reasons for complete denture failures is the inaccurate determination of OVD^5^. In clinical practice, there are a variety of methods but none of them can provide an exact measurement due to a vast range of variations among individuals^6^. The methodologies prior to extractions include measurement of intraoral dimensions^7^, tracings of the facial profile^8^, pre-extraction photographs^9^, phonetics^10^, cephalometric approaches^11^, and measurement with an oro-facial device^12^. Patient’s pre-extraction photographs may pose difficulties in recording OVD because of aging and loss of facial height^13^, hence the clinician must think likely about using post-extraction methods. They include assessment of physiologic rest position^14^, facial esthetic appearance^15^, deglutition method^16^, evaluation of the former dentures if the OVD is acceptable^17^, measurement of biting force^18^, the use of magnetic plates^19^. The changes in appearance of lower one-third of the face can be comprehended in patients with good skin and muscle tone and with advancing age this may not deem reliable^20^.

The idea of using anthropometric measurements came from Leonardo Da Vinci drawings which have further been explored by various researchers^21^. Anthropometric techniques are non-invasive, simple, low-risk and most of all inexpensive and straightforward to carry on^22^. In a study on dentate individuals, it was found that the length of the index finger (2D) was found to be almost equal to the occlusal vertical dimensions measured from the base of the nose to the base of the chin (Sn–Me)^23^. A similar study on dentate Sudanese women, indicated correlations between different finger lengths, namely the index finger (2D), the ring finger (4D) and little finger (5D) with the OVD^24^. There have been established studies on dentate individuals that the length of index finger (2D) has strong correlation with the OVD in males while length of little finger (5D) has strong correlation with the OVD in females in different populations^25–27^. The length of the thumb can be fundamentally correlated for OVD measurements when compared with the distance from rima oris to pupil of the eye^28^, as well as the eye-ear distance and pupil of the eye to rima oris^29^. The length of the palm and four fingers width^30^, and 5D corresponds with patient’s OVD when compared with chin-nose distance^31,32^. Rodríguez found similarity between the 2D, 3D, 5D to the OVD^33^. However, further exploration is needed in edentulous patients through a clinical trial since measurement of accurate OVD constitutes to play an important role in overall patient satisfaction with complete dentures^34^. There are several factors leading to acceptability of complete dentures, evaluation has been done to find out the denture satisfaction among conventional complete denture wearers after reduction of OVD^35^. However, due to lacuna in previous literature, there is no method to predict the denture satisfaction and quality of life with dentures in completely edentulous patient^36^.

Recording of the OVD is a tedious step in constructing complete dentures, and the limiting factor in using conventional approaches is that it depends on unreliable and inconsistent measurements of facial landmarks which vary with advancing age^37^. In such circumstances, there is a need to develop an alternate, consistent, and more reliable method for measuring near-ideal OVD^38,39^. It is worthy to mention that patients present for complete dentures most commonly without any pre-extraction records. The only possibility for an accurate calculation for OVD is to focus on post-extraction methods, out of which anthropometric measurements of facial landmarks can be most relied upon. Since length of the index finger remains constant throughout life after adolescence, it may provide an easy, less time-consuming alternative for recording the lost OVD^40^. Hence, keeping in account this hypothesis the present clinical trial was conducted on edentulous patients in the effort to apply the anthropometric methods to correlate the length of the index finger of right hand (2D) using a vernier caliper to measure the OVD from the base of the nose (Subnasion-Sn) to the base of the chin (Menton-Me) using the Willis Gauge. Therefore, the first null hypothesis would mean no correlation exists for the VDO being recorded from Sn–Me and length of the index finger (2D). The second objective was to assess the patient satisfaction through a Visual Analog Scale (VAS) scale and comparing both the methods. Therefore, the second null hypothesis would mean a dissatisfaction. This study seeks to bridge the research gap of limited published clinical trials on edentulous patients and helps clinicians to adapt to an alternate methodology to measure the physiologic OVD. Furthermore, having a complete denture with near-ideal re-establishment of OVD would result in greater patient satisfaction.

## Methods

### Research design and settings

The research study was a randomized controlled trial (RCT), conducted at the Department of Prosthodontics at Dow University of Health Sciences following the CONSORT guidelines^41^ for parallel group randomization. The trial was approved at the registry of https://clinicaltrials.gov/ under the protocol identifier: NCT05153213, registered on 10/12/2021. Ethical approval was acquired from the Institutional Review Board of DUHS (reference no. IRB-1189/DUHS/Approval/2019/32).

### Sample estimation and eligibility

**Figure 1:**
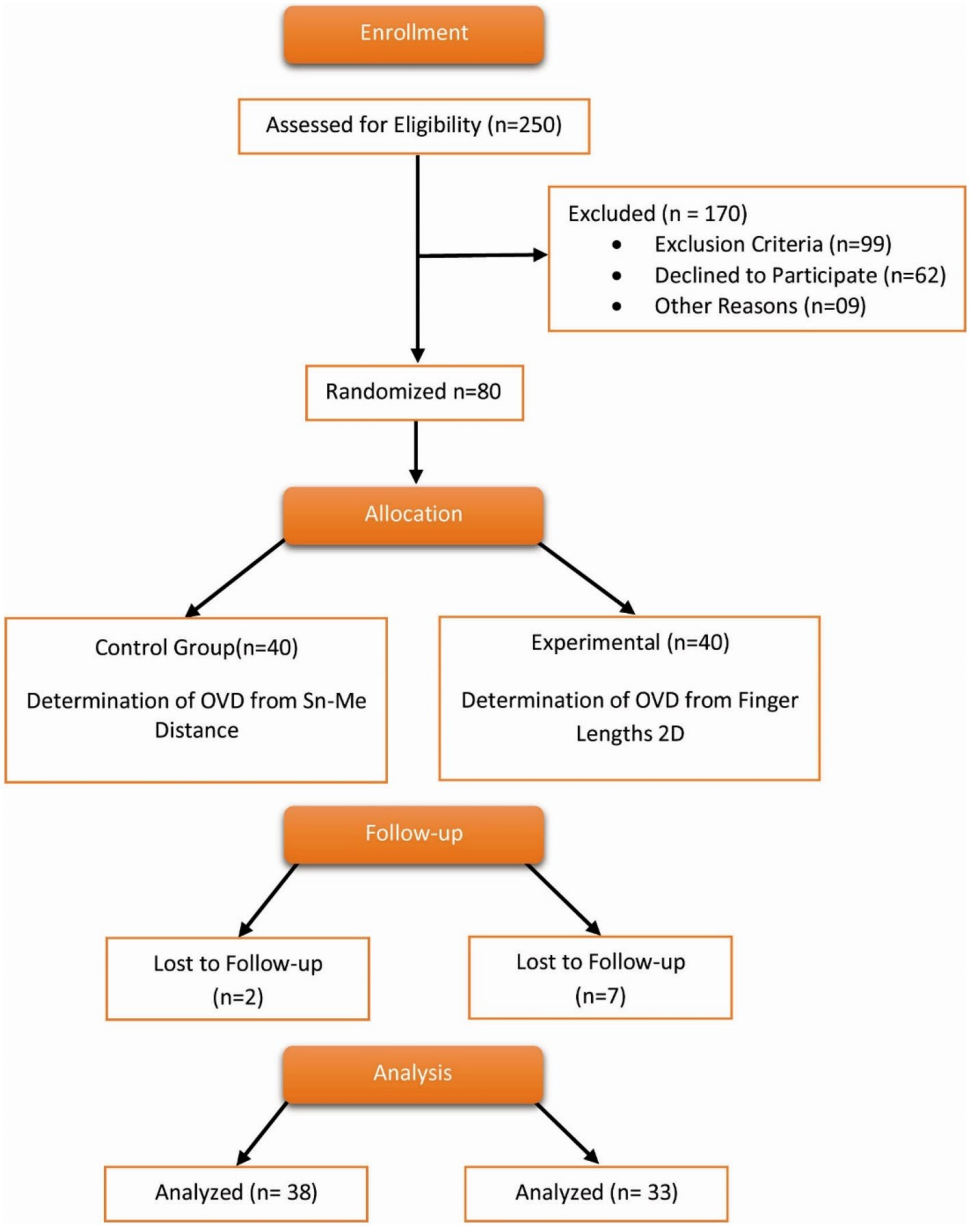
CONSORT participant flow chart.

The studies regarding the measurement of the length of 2D involved dentate individuals but none included edentulous patients or complete dentures fabrication^23,24,26^. As per the literature, a sample size of 30 is reported to be sufficient in the case of experimental trials. For the purpose of our study, we recruited 80 participants fulfilling our eligibility criteria^42^. Since our outcome variable is VAS, which is a continuous variable, therefore a sample size of 80 patients fulfilling the criteria was recruited in the study. Collectively 250 patients were selected for the study by the principal researcher, out of which 170 were excluded and 80 were randomized for the study. Patients were allocated in two parallel groups; experimental and control, and after losing 9 patients to follow-up, 71 patients completed the trial, 38 in control group and 33 in experimental. The study participants were randomized after they met with the eligibility of being completely edentulous both male and female, aged between 30 and 80 years, males without a beard and patients willing to participate. A consent form was explained and signed by the patients. Partially dentate patients, patients with any maxillofacial or myofascial disorders or history of orthognathic surgery, deformities or disfigurement of fingers, neurological problems in the head and neck, any bony defects or visible sharp spicules, nose, or chin deformity, TMJ disorders, or having bulky chin area were excluded from the study. Randomization was done by the principal investigator using a coin toss method where patients were equally divided into experimental and control groups and were kept blind to the treatment assigned. Only the principal investigator knew that a coin toss of heads would receive anthropometric method (experimental group) and tails would receive conventional method of recording OVD (control group). (Fig. 1).

### Data collection method

#### Measurement of distances

The clinical trial started with the procedure for their respective complete dentures till the end of the prosthesis fabrication and follow-ups done 1 week after insertions. Before proceeding with the facial and measurement of 2D at the appointment for jaw relation records for both parallel groups, verbal instructions were given to the patients, and a written informed consent form was obtained by the participants for the research.

A digital vernier caliper (Kangalu; Shenzhen Ruizh Industrial Co., Ltd., Shanghai, China) was used to measure the lengths of the right 2D in the experimental group. All measurements were made on the right hand having asked the patients to have their nails trimmed^43^. The length of 2D was recorded from the tip of the finger to the root (crease) which coincides with the metacarpophalangeal joint and having the hand in a supine position placed against a flat surface. A Willis gauge (SHS Healthcare Industries, Sialkot, Pakistan) was used to record the distance between the base of the septum of nose (Sn) to the base of chin (Me), used as control. The patient was seated comfortably and upright on the dental chair with the head unsupported. The posterior occlusal plane orientation was adjusted to coincide with the ala-tragus line (Camper line) using a Fox plane (Yonghao Industries, Jiangsu, China). The bite blocks were placed in the oral cavity during the clinical procedure for establishing jaw records^44^.

**Figure 2:**
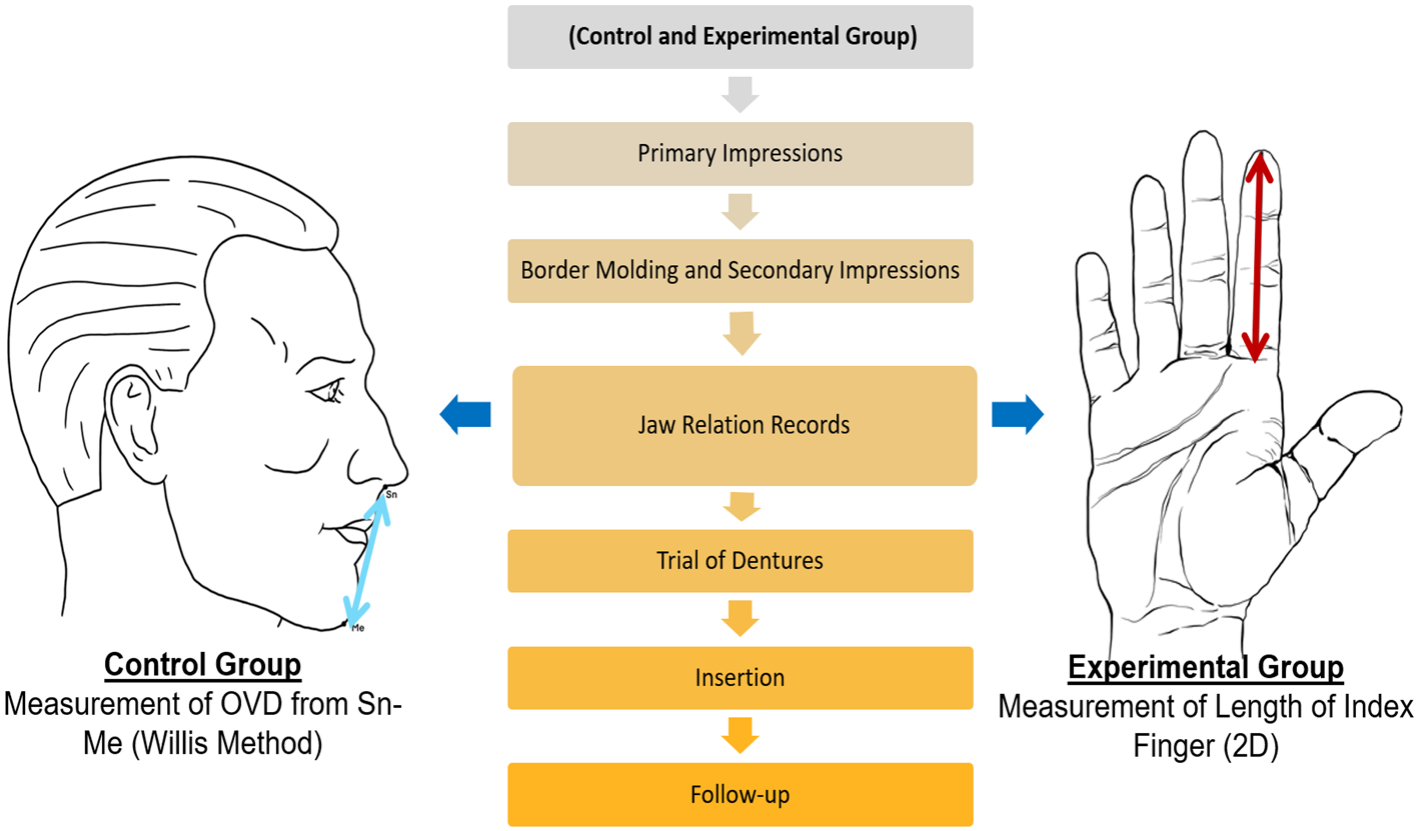
Flowchart of sample collection.

Dentures were fabricated on the OVD obtained from both experimental and control groups. For experimental methods index finger length, having the most statistical co-relation to the distance from Sn–Me was used for determining the OVD to achieve the first outcome of study. Denture fabrication was performed as per the standard protocols of the textbook^4^. Patients were actively involved in denture fabrication especially teeth selection and positioning so that the ownership of dentures can be improved. They were instructed before and during denture fabrication to not to wear their previous prosthesis for at least 2 h prior to the procedure, in case of ill-fitting dentures, patient was discouraged to use them. Clinical procedures and all laboratory work leading to fabrication and deliverance of dentures had been carried out in the Department of Prosthodontics by the principal researcher himself. A flow-chart of the sample collection is provided in Fig. 2.

#### Denture satisfaction measurement using Visual Analog Scale (VAS)

The patients were recalled for denture insertions and all necessary adjustments made prior to insertion. Later, they were asked for their responses regarding their dentures’ satisfaction after a 1-week follow-up and marked according to the VAS^2,45^, used as a dependent variable in the study. The questionnaire included questions such as, whether the patient is consistent in wearing the prosthesis, the comfort while wearing dentures, if there’s pain during mastication with dentures, swallowing food and water while wearing dentures, if the patient experiences gagging, if the patient is satisfied with the facial appearance, level of satisfaction with the function of denture, whether the patient feels the need to get the prosthesis replaced.

### Statistical analysis

The data collected was subjected to statistical analysis using statistical software (IBM^®^ SPSS^®^ Statistics version 16.0). In descriptive statistics, percentage and frequency were reported for categorical variables like gender and patients’ group whereas mean and standard deviation were reported for continuous variables like age (years) and length (mm). The assumption of normality was checked by using the Shapiro–Wilk test of the continuous variables. Mann–Whitney U test was applied to check mean differences for distance Sn–Me (mm) and length 2D (mm) between male and female. Spearman’s rho correlation coefficient was computed to check the strength of the relationship between distance Sn–Me and length of index finger (2D) for males and females separately. A Fisher’s exact test was run to check the association between categorical variables and satisfaction level regarding dentures. All test results having *p*-values less than or equal to 0.05 level of significance were considered statistically significant.

### Ethics approval and informed consent

This study and clinical trial was approved by the Institutional Review Board of Dow University of Health Sciences (reference no.IRB-1189/DUHS/Approval/2019/32). All methods were carried out in accordance with relevant guidelines and regulations (declarations of helsinki). A written informed consent was obtained from the study participants.

### Consent for publication

A written informed consent for publication was also included from the participants.

## Results

### Study sample characteristics

A total of 80 participants were initially recruited and randomized into two parallel groups, with forty in each group. Out of which 71 completed the trial and were statistically analyzed after lost to follow up, 35.2% (n = 25) were males and 64.8% (n = 46) were females. There were 53.5% (n = 38) patients who were in control group and 46.5% (n = 33) patients belonged to experimental group. The mean (± SD) age of the patients was 58.2 (± 7.4) years and ranged between 45 and 77 years. (See Table 1).

**Table 1:** Baseline characteristics of the participants.

Characteristics	n = 71	%
Gender		
Male	25	35.2
Female	46	64.8
Group		
Control	38	53.5
Experiment	33	46.5

	Mean ± SD	Range
Age (years)	58.2 (± 7.4)	45–77

*SD* Standard deviation.

### Mean comparison

In Table 2, the mean differences for distance Sn–Me (mm) and length of 2D (mm) between male and female can be interpreted. It was noted that all length measurements are greater among males as compared to females. Distance Sn–Me and measurement of 2D were found to be statistically significantly different between males and females.

**Table 2:** Mean comparison of distances Sn–Me (mm) and finger length (mm) by gender.

Variables	Male	Female	*p*-value^a^
	Mean ± SD		
Distance-Sn–Me	69.73 ± 6.83	68.36 ± 3.94	0.010
Length-2D	70.12 ± 7.08	68.97 ± 3.72	0.023

*SD* Standard deviation.

^a^Mann–Whitney U test; *p*-values were considered significant at 0.05.

### Gender correlation

The strength of the relationship between distance Sn–Me (mm) and length of fingers (mm) for males and females was computed separately using Spearman’s correlation co-efficient $r_{s} = 1 - \frac{{6\sum {d_{i}^{2} } }}{{n(n^{2} - 1)}}$. In both genders, strong, positive, and statistically significant correlations were found between these measurements. Among females’ highest correlation was found between distance Sn–Me (mm) and length 2D (r = 0.966, *p*-value =  < 0.001). (See Table 3).

**Table 3:** Correlation between distance-Sn–Me (mm) and Length-D (mm) stratified over gender.

Variables	Distance-Sn–Me (mm)			
	Male		Female	
	r	*p*-value^a^	R	*p*-value^a^
Length-2D	0.959	< 0.001	0.966	< 0.001

r, correlation coefficient.

^a^Spearman’s rho correlation; *p*-values were considered significant at 0.05.

### Denture satisfaction

**Figure 3:**
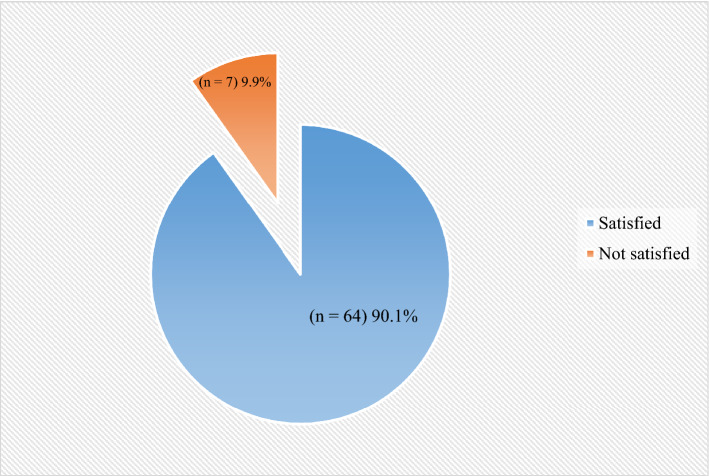
Satisfaction level regarding dentures.

VAS was used to assess the satisfaction level of the patients through a questionnaire. There was a total of 8 questions asked (scores range: 0–10 each) to measure satisfaction level were added to calculate final satisfaction scores; it ranged from 0 to 80. The cutoff point was 40. It was found that 90.1% (n = 64) of patients were satisfied with complete dentures as represented in Fig. 3.

While checking the association between categorical variables and satisfaction level regarding dentures it was found that among males 88.0% were satisfied with complete dentures whereas among females 91.3% were satisfied. It was also noted that 84.2% patients from the control group and 97.0% patients from the experimental group were satisfied with the use of their dentures. However, no variable was found to be statistically significantly associated with the satisfaction level. (See Table 4).

**Table 4:** Association between satisfaction level and other covariates.

Variables	Satisfaction level		*p*-value^a^
	Not satisfied (n = 7)	Satisfied (n = 64)	
	n (%)		
Gender			
Male	3 (12.0)	22 (88.0)	0.691
Female	4 (8.7)	42 (91.3)	
Age (years)			
≤ 55	2 (6.2)	30 (93.8)	0.446
> 55	5 (12.8)	34 (87.2)	
Group			
Control	6 (15.8)	32 (84.2)	0.113
Experiment	1 (3.0)	32 (97.0)	

^a^Fisher exact test; *p*-values were considered significant at 0.05.

## Discussion

This clinical trial was done in pursuit of a reliable and less time-consuming technique to restore the lost OVD for the fabrication of complete dentures and to find out a practical solution for the patient and the dentist by measuring the length of the index finger and correlating it with the distance measured from the base of the nose (Sn) to base of the chin (Me) and to later assess denture satisfaction by comparing both the methods. The present study was carried out on edentulous patients and most of the patients were females, average age of the patients was 58.23 years and the mean values for length of index finger in males was measured to be 70.12 ± 7.08 and in females 68.97 ± 3.72. The index finger selected was of the right hand as there are no differences in symmetry, and this uniform the method for measurements^43^. Our results demonstrating males exhibiting longer finger length measurements than females is attributed to gender dimorphism because of androgen exposure levels at puberty^46^. The present study established that in both genders, strong, positive, and statistically significant correlations were found between VDO being recorded from Sn–Me and length of the index finger (2D) measurements hence the first null hypothesis was rejected. However, the distances Sn–Me and all measurements of 2D, were found to be statistically significantly different between both the genders and males demonstrating higher level of measurements, this is in agreement with a previous study, where significant correlation was found between the OVD and length of 2D^23^. Similar results were reported^26^, 2D has strong correlation in males, the mean length being 71.6 mm while length of little finger (5D) has a strong correlation in females both having teeth present, the length of the little finger does not match with the current study. One study concluded the mean OVD for males being 70.76 mm similar to that of the current study which is 69.73 mm, and for females 64.54 mm in contrast to the current study’s female population because of apparent anthropometric variations in race and ethnicity^25^. A significant correlation between the length of each of these fingers (2D, 4D and 5D) and the OVD measured from distances Sn–Me as well as N–Gn. The mean length of 2D was measured to be 78.28 mm, since the participants were Sudanese and dentate, the results did not match our study’s gender and population^24^.

In harmony with the present study, no significant differences between the OVD measured from Sn–Me to the length of 2D. However the mean values of OVD and 2D measurements (78.4 mm for dentate and 83.2 mm for edentulous individuals) did not correlate with the distances recorded for our study^47^. Similar studies by^30,33^ used this method for establishing OVD, the recordings were different from our study population but in agreement with the current study strong correlation was found between the facial and finger measurements. A study on the Kashmiri population has also found an association between the vertical dimensions and 2D for males, however, a dissimilarity in values and absence of patient’s denture satisfaction since the participants were dentate individuals^27^.

It is vital to remember that all the comparable as well as contrasting studies mentioned have suggested the finger length method for the measurement of correct OVD for future projections. The dissimilar results, however, are due to the differences in gender and demographic ethnicity and involvement of younger dentate participants.

The current study’s second objective was to evaluate denture satisfaction regarding the determination of OVD using the length of 2D of the right hand after 1 week of follow up. Collectively 90.1% (64 out 71) from both groups and a total of 97% patients from the experimental group were satisfied with their complete dentures, this rejects the second hypothesis. The questions asked were pertaining to an increase or decrease in the OVD. The responses asked from the patients were the regular use of dentures, comfort during chewing, pain during chewing, ease during swallowing, gagging, appearance, satisfaction with function, and replacement of denture, and the responses were recorded with a VAS questionnaire^2,45^.

Previously a VAS questionnaire was used to record the responses regarding complications in denture wearers and it was communicated that loss of denture retention was the most prevalent complication, while the assessed the vertical dimensions which were graded as normal, high, or low, and upon examination, it was found out 69.2% of patients had low vertical dimensions. However, the study did not assess the satisfaction regarding the measurement of OVD through anthropometry as done in this current study^2^. Sato et al. quantified the overall satisfaction of complete denture patients through chewing, speech, pain in the lower jaw, aesthetics, fit of the upper prosthesis, retention for lower arch, and comfort for the upper arch. The factors which weren’t quantified were satisfaction associated with the OVD^48^. Alternatively, in another study evaluation was done to find out the denture satisfaction among conventional complete denture wearers after reduction of the vertical dimension of occlusion and it was found that there were significant differences in satisfaction levels between suitable and reduced OVD and the patients found more comfort in using complete dentures which had reduced OVD^35^.

There is inadequate research on data such as vertical dimension, centric relationship, and location of artificial teeth relative to denture foundation and underlying tissues that provide knowledge about the general pattern of denture quality provided in general practice. There is also a lack of quality studies to determine a prognostic preoperative method for forecasting the acceptability of denture. Hence, there is no gold standard to predict denture satisfaction and quality of life with dentures in a completely edentulous patient.

This current study signifies that the index finger of the right hand can be used for the assessment of vertical dimension of occlusion in edentulous patients seeking complete dentures which will result in improved denture satisfaction. The study will also help in bridging the knowledge gap among clinicians to adapt to a different methodology which is relatively easy and straightforward procedure giving reliable outcome for measurement of lost OVD.

The clinical significance of this study can be emphasized by the fact that this was a person-centered clinical trial where the procedure was easy, non-invasive, less time-consuming, and economical without having the necessity for any sophisticated equipment for radiography nor any specialized measuring tools. The study also provides insights that a simple and inexpensive measuring instrument (a vernier caliper), can be used for anthropometric recordings and would deliver the accuracy required.

According to the study's limitations, a small number of edentulous patients were randomized for the trial and because the data was limited to the Pakistani population, racial and ethnic disparities must be considered when comparing the results. Also, only 1 week follow-up was taken for denture satisfaction from the participants, other concerns were not addressed that could be reported during further follow-ups. The impact of the impression technique, occlusal scheme for teeth setup was not included in the parameters for denture satisfaction.

In response to this study, more extensive clinical trials need to be conducted making this technique more effective for routine procedures and further research is recommended signifying the comparison among different anthropometric techniques for future reference.

## Conclusion

Measuring the length of the index finger of the right hand can be an adjunct method for the determination of lost OVD for edentulous patients requiring complete dentures since strong statistically significant correlations were found between length of 2D and distance Sn–Me. This method can be used use in low-resource settings, having lack of access to sophisticated measuring dental and radiographic equipment.

## Data Availability

All datasets used and analyzed in this study are available from the corresponding author on request.

## Abbreviations

OVD: Occlusal vertical dimensions
Sn–Me: Distance from septum of the nose (Sn) to Menton—base of chin (Me)
VAS: Visual Analog Scale
